# Clinicopathological features and prognosis of TFE3-positive renal cell carcinoma

**DOI:** 10.3389/fonc.2022.1017425

**Published:** 2022-10-06

**Authors:** Xiang Dong, Yuxin Chen, Jun Pan, Wenliang Ma, Peng Zhou, Ming Chen, Hongqian Guo, Weidong Gan

**Affiliations:** ^1^ Department of Urology, Affiliated Drum Tower Hospital, Medical School of Nanjing University, Nanjing, China; ^2^ Department of Urology, Nanjing Drum Tower Hospital Clinical College of Nanjing Medical University, Nanjing, China; ^3^ Department of Pathology, Affiliated Drum Tower Hospital, Medical School of Nanjing University, Nanjing, China

**Keywords:** TFE3, Xp11.2 translocation, RCC (Renal Cell Carcinoma), Clinico pathologic characteristics, prognosis

## Abstract

**Background:**

This study aimed to investigate the expression profile of TFE3 in renal cell carcinoma (RCC) and the clinicopathological features as well as prognosis of TFE3-positive RCC.

**Methods:**

Tissue sections from 796 patients with RCC were collected for immunohistochemical staining of TFE3. Molecular TFE3 rearrangement tests were also carried out on the TFE3-positive RCCs using fluorescence *in situ* hybridization and RNA-sequencing assays. Both clinicopathological features and follow-up information were collected for further analysis.

**Results:**

The present study showed that 91 patients with RCC (91/796, 11.4%) were TFE3 positive expression but only 31 (31/91, 34.1%) of the patients were diagnosed with Xp11.2 translocation RCC. Further, it was found that the patients with TFE3-positive RCCs were more likely to develop lymph node and distant metastasis at diagnosis as well as presented a significantly higher WHO/ISUP nuclear grade and AJCC stage as compared with patients with TFE3-negative RCCs (*p*<0.01). Results of univariate and multivariate analyses showed that TFE3 positive expression was an independent prognostic factor associated with poor progression-free survival. Further, the findings of survival analysis showed that patients with positive TFE3 expression showed a shorter progression-free survival as compared with the patients with negative expression of TFE3 (*p*<0.001). In addition, results of the survival analysis found that there was no significant difference in progression-free survival between the Xp11.2 translocation RCC and TFE3-positive non-Xp11.2 translocation RCC groups (*p*=0.9607).

**Conclusion:**

This study found that nuclear TFE3 expression is not specific to the Xp11.2 translocation RCC. Moreover, the positive TFE3 expression is associated with tumor progression and poor prognosis in patients with RCC irrespective of the presence of TFE3 translocation.

## Introduction

The TFE3 gene which is located on the chromosome band Xp11.2 belongs to a member of the microphthalmia transcription family (MiTF) (1). The MiTF family of genes plays crucial role on autophagy, lysosome generation, and have been involved in the progression of various tumors (2, 3). When cells encounter hypoxia or starvation, it has been found that the TFE3 proteins translocate from cytoplasm into the nucleus (4, 5). It has been shown that the translocation of TFE3 genes may result in fusion of the TFE3 gene with other partner genes (6–8). This leads to expression of TFE3 proteins. Translocation of TFE3 and expression of TFE3 proteins can be identified in different types of tumors, such as Xp11.2 translocation renal cell carcinoma (RCC), epithelioid haemangioendothelioma, alveolar soft part sarcoma, perivascular epithelioid cell tumor, ossifying fibromyxoid tumor, and malignant chondroid syringoma (2). Xp11.2 translocation RCC has been shown to be the most representative tumor among the mentioned tumors. The Xp11.2 translocation RCC was first recognized as a separate entity by the World Health Organization (WHO) in 2004. It was then reclassified as a member of the MiTF translocation RCC in 2016. The tumor accounts for approximately 30% of pediatric RCCs and between 1 and 5% of adult RCCs (9–11). In adults, the tumor (Xp11.2 translocation RCC) remains a rare disease with an invasive course and poor prognosis (7, 11–13).

With the advancement of research, several studies have shown that even tumors lacking TFE3 translocation still show nuclear TFE3 immunoreactivity. This include solid pseudopapillary neoplasm of the pancreas, granular cell tumor, and ovarian sclerosing stromal tumor (2, 14). The RCCs with positive expression of TFE3 but without translocation have also been reported. This is an indication that not all TFE3-positive RCCs that belong to Xp11.2 translocation RCCs (7, 11, 12, 15, 16). However, available information regarding the characteristics of such RCCs remain rare. Meanwhile, the current studies showed that positive expression of TFE3 was associated with invasive course and poor prognosis in patients with RCC which was similar to that of patients with Xp11.2 translocation RCC (12, 17). This is the first study to report on the expression of TFE3 in such a relatively large cohort of RCCs. Furthermore, the present study also investigated clinicopathological features and prognosis of patients with TFE3-positive RCCs.

## Materials and methods

### Patient selection

A total of 796 adult patients with RCCs who had undergone radical or partial nephrectomy in Nanjing Drum Tower Hospital were reviewed between January 2018 and September 2021. The inclusion criteria were (1): pathologically confirmed RCCs (2); complete clinicopathological and follow-up information (3); enough tumor specimens for further analysis. Immunohistochemical (IHC) examination for TFE3 was conducted in all selected patients. Fluorescence *in situ* hybridization (FISH) assay was performed on all the TFE3-positive patients to confirm the diagnosis of Xp11.2 translocation RCC. For cases with NONO-TFE3, GRIPAP1-TFE3, RBX-TFE3, and RBM10-TFE3 rearrangement which was caused by the X-chromosome inversion, there were previous studies that showed the cases have strong positive TFE3 IHC staining but equivocal split signals (18–20). The cases in this study with moderate or strong nuclear TFE3 immunoreactivity were included for further RNA-sequencing. This was in consideration of potential false negative cases with equivocal split signals. Clinicopathological and survival data were also collected for every patient, including age, gender, maximum tumor diameter, tumor location, WHO/ISUP (World Health Organization/International Society of Urologic Pathology) nuclear grade, AJCC (American Joint Committee on Cancer) stage, and follow-up information. Remarkably, the WHO/ISUP grading system was found not applicable for the Xp11.2 translocation RCC and chromophobe RCC (21). The approval of the present retrospective study was provided by the institutional review board of Nanjing Drum Tower Hospital and the informed consents was waived from the selected patients in the current study.

### Immunohistochemistry

Immunohistochemistry of TFE3 were performed on the four-μm-thick formalin-fixed paraffin-embedded (FFPE) sections of all the RCC cases. IHC staining of the sections was carried out using anti-TFE3 antibody (SC-5958,1:300; Santa Cruz, CA). Further, IHC assay of the TFE3 was conducted though labeled streptavidin-biotin method followed by overnight incubation (22). The Xp11.2 translocation RCC and IgG were used as positive and negative controls for the IHC assay, respectively. Only nuclear TFE3 staining was considered as the positive result. The final result was analyzed by two independent observers and the inconsistent result was resolved by an experienced pathologist. To perform a semi-quantitative assessment, the results of this study were analyzed in reference to the previously reported intensity and degree of nuclear staining (22, 23). The IHC score was calculated by multiplying the percentage of positive cells (0–100) by the staining intensity (0=no staining, 1 = weak staining, 2 = moderate staining, and 3 = strong staining). For the result of immunostaining, a score of between 0 and 25 was considered negative (–), a score of between 26 and 100 was considered weak (+), a score of between 101 and 200 was considered moderate (++), and a score of between 201 and 300 was considered strong (+++).

### TFE3 break-apart FISH

In the present study four-μm-thick FFPE tissue sections were prepared for FISH assay using dual-color break-apart TFE3 Probes (LBP, Guangzhou, China). Fluorescence signals were analyzed using an Olympus BX51TRF fluorescence microscope (Olympus, Tokyo, Japan) with a triple emission filter (DAPI/FITC/Texas Red) and the FISH assay analysis software (Imstar, Paris, France). Further, at least 100 non-overlapping nuclei were counted in every sample. A minimum of 100 tumor nuclei were analyzed through fluorescence microscopy, and the split signal was defined as the distance >2 signal diameter. In addition, positive result of the FISH assay was defined as more than 10% of tumor nuclei with the split signal. In male patients, the positive results consisted of a single pair of separated red and green signals. In female patients, the positive results consisted of a fused signal pair (yellow) and an additional pair of split signals.

### RNA-sequencing

Total RNA was first extracted from the sections of FFPE tissue using the RNeasy kit (Qiagen, Hilden, Germany) according to instruction provided by the manufacturer. RNase H enzyme was used to deplete ribosomal RNA. KAPA Stranded RNA-seq Kit containing RiboErase enzyme (HMR) (KAPA Biosystems) was then used for preparation of the library. In addition, the quality of library was assessed using an Agilent Bioanalyzer 2100 system (Agilent, USA). Final libraries were subjected to a high-throughput Illumina HiSeqTM 2000 platform (California, USA), which was conducted by the GloriousMed Technology (Beijing, China).

### Statistical analysis

Statistical analyses of the data obtained in the current study was conducted using SPSS 23.0 software (Chicago, IL, USA). The Kaplan-Meier survival curves were drawn using GraphPad Prism 8.0 (GraphPad Software, USA). Evaluated characteristics were compared among the three groups using the Mann-Whitney U test or χ^2^ test. Survival analysis was performed using the Kaplan-Meier survival curves and log-rank tests were used to compare the drawn Kaplan-Meier survival curves. Cox proportional hazards regression model was conducted for both univariate and multivariate analysis. Statistical significance was set at *P* value less than 0.05.

## Results

The RCCs in the present study were divided into two groups according to the positive and negative expression of TFE3. The clinicopathological characteristics were then compared between the two groups (**Table 1**). It was evident that there was a significant difference in the median age of patients between the TFE3-positive and negative RCC groups (*p*<0.001). Results of the present study showed that the patients with TFE3-positive RCCs were significantly more likely to develop lymph node and distant metastasis at diagnosis as compared with the patients having TFE3-negative RCCs (*p*<0.001). Furthermore, it was noted that the TFE3-positive RCC group was significantly associated with a higher WHO/ISUP nuclear grade and AJCC stage as compared with the TFE3-negative RCC group (*p*<0.01).

**Table 1:** Clinicopathological characteristics of the 796 patients with RCC.

	TFE3-positiveRCC			TFE3-negative RCC	P	P*
	Total(n=91)	Xp11.2 translocation RCC (n=31)	Non-Xp11.2 translocation RCC (n=60)	(n=705)		
Median age (range, years)	54 (19–77)	43 (24-74)	59 (19-77)	58 (23-90)	<0.001	<0.001
Gender					0.048	0.149
Male	54	14	40	472		
Female	37	17	20	233		
Median tumor size (range, cm)	4.5 (1.1-16.5)	4.4 (2.2-16.5)	4.5 (1.1-12)	3.9 (0.7-19.5)	0.235	0.056
Tumor location					0.242	0.555
Left	51	20	31	372		
Right	40	11	29	333		
Lymph node metastasis					0.084	<0.001
YES	10	6	4	9		
NO	81	25	56	696		
Distant metastasis					0.405	<0.001
Yes	6	3	3	6		
No	85	28	57	699		
WHO/ISUP grade					NA	<0.001
1+2	24	NA	24	518		
3+4	33	NA	33	153		
AJCC stage					0.406	0.002
I+II	72	23	49	634		
III+IV	19	8	11	71		
Histopathological subtype of RCC					NA	NA
Clear cell	45	0	46	626		
Papillary	12	0	11	45		
Chromophobe	3	0	3	15		
Xp11.2 translocation	31	31	0	0		
Others	0	0	0	19		

RCC, renal cell carcinoma; NA, not available; P, TFE3-positive Xp11.2 translocation vs non-Xp11.2 translocation RCC cases; P*, TFE3-positive vs TFE3-negative RCC cases; Bold values indicate P<0.05.

A total of 91 TFE3-positive RCCs were identified in the current study. Results of a comparative analysis of the clinicopathological characteristics between Xp11.2 translocation RCCs and TFE3-positive non-Xp11.2 translocation RCCs were also as shown in **Table 1**. It was evident that the patients with Xp11.2 translocation RCCs were statistically younger (median age, 43) as compared with patients with TFE3-positive non-Xp11.2 translocation RCCs (median age, 59) (*p*<0.001). In addition, it was found that the patients with Xp11.2 translocation RCCs were more frequently female predominated (55%) as compared with the patients with TFE3-positive non-Xp11.2 translocation RCCs (33%) (*p*=0.048). Further, the results of the present study showed that there were no significant differences in the median tumor size, tumor location, lymph node metastasis, distant metastasis, and AJCC stage between the two studied groups. According to the WHO/ISUP grading system, 33 out of 57 patients with TFE3-positive non-Xp11.2 translocation RCCs were at grade III or IV.

**Figure 1:**
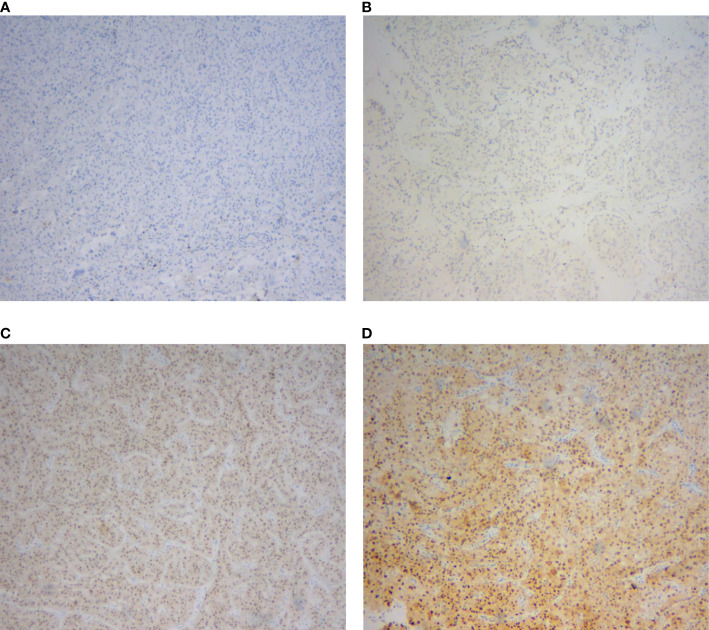
Immunohistochemical staining of TFE3 protein in renal cell carcinomas. **(A)** Negative expression. **(B)** Weak expression. **(C)** Moderate expression. **(D)** Strong expression.

**Figure 2:**
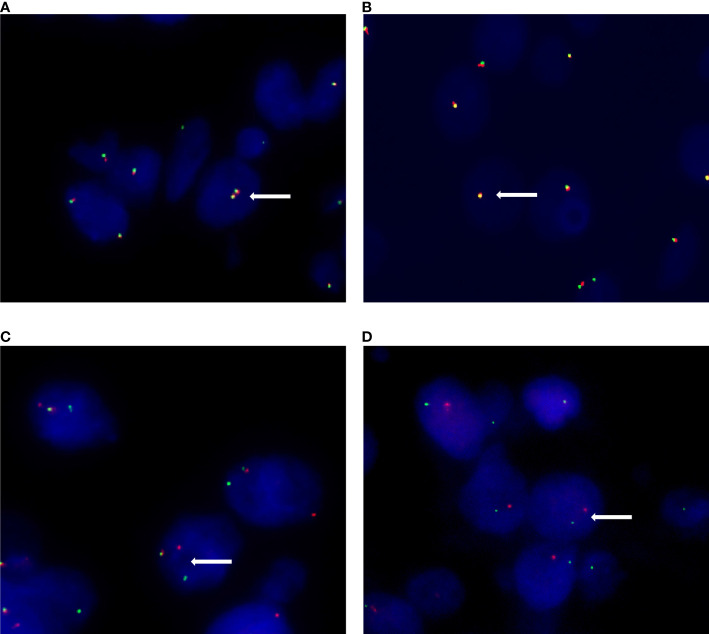
Different signal patterns in FISH staining. **(A)** Female patients show two pairs of fused (yellow) signal in non-Xp11.2 translocation renal cell carcinomas. **(B)** Male patients show one fused (yellow) signal pair in non-Xp11.2 translocation renal cell carcinomas. **(C)** Female patients show one fused (yellow) signal pair and an additional pair of split (red and green) signals in Xp11.2 translocation renal cell carcinomas. **(D)** Male patients show one signal pair of seperated red and green signals in Xp11.2 translocation renal cell carcinomas.

**Figure 3:**
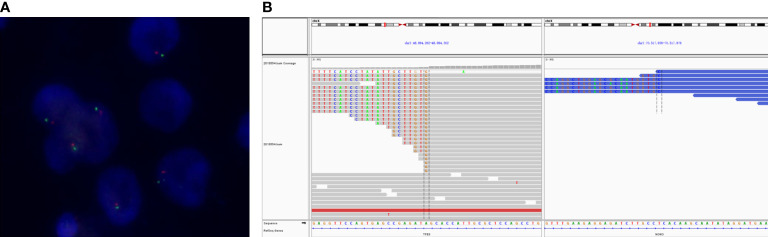
The typical FISH and RNA-sequencing results of NONO-TFE3 renal cell carcinoma. **(A)** FISH shows a single pair of separated red and green signals but remains in close proximity. **(B)** A fusion of TFE3 (doner end: chrX: 48894302) and NONO (acceptor start: chrX: 70517936) was detected in the tumor visualized in the Integrative Genomics Viewer.

A total of 91 RCCs showed positive expression of TFE3, including 56, 18, and 17 cases with weak, moderate, and strong expression, respectively. All of the 17 strong expression cases belonged to patients with Xp11.2 translocation RCCs. Among the 56 weak expression cases, only 6 cases were confirmed to be from Xp11.2 translocation RCC group (**Table 2**). Typical images of the IHC staining for both TFE3 and FISH were as shown in **Figures 1**, **2**, respectively. For further analysis, RNA-sequencing was performed on the cases with moderate or strong TFE3 expression. Ultimately, two TFE3-positive RCC cases with equivocal split signals were detected using FISH assay and were further diagnosed with NONO-TFE3 RCC by RNA-sequencing in the cases. The obtained results of the typical FISH assay and RNA-sequencing were as shown in **Figure 3**.

**Table 2:** Correlation of IHC and FISH.

	TFE3 IHC staining			N
	Weak (+)	Moderate (++)	Strong (+++)	
FISH-positive	6 (10.7%)	8 (44.4%)	17 (100%)	31
FISH-equivocal	0 (0%)	2 (11.2%)	0 (0%)	2
FISH-negative	50 (89.3%)	8 (44.4%)	0 (0%)	58
Total	56	18	17	91

IHC, immunohistochemistry; FISH, Fluorescence in situ hybridization.

Univariate and multivariate Cox proportional hazard model were performed in the present study to clarify the risk factors of progression-free survival. Results of the univariate analysis study showed that age, AJCC stage, and expression of TFE3 were significantly correlated with progression-free survival (*p*<0.05). On the other hand, the obtained results of multivariate analysis showed that higher AJCC stage (Hazard Ratio, HR=11.4; 95% Confidence interval, CI 7.463-17.414; *p*<0.001) and positive expression of TFE3 (HR=2.32; 95% CI 1.344-4.000; *p*=0.002) were the independent prognostic factors associated with poor progression-free survival (**Table 3**).

**Table 3:** Results of univariate and multivariate analysis for progression-free survival.

	Univariate analysis		Multivariate analysis	
Factors	HR (95% CI)	P	HR (95% CI)	P
Age				
≤50 years >50 years	Reference 2.35 (1.236-4.469)	0.009	1.691 (0.862-3.315)	0.126
Gender				
Male Female	Reference 1.016 (0.622-1.659)	0.949		
Tumor location				
Left Right	Reference 0.926 (0.584-1.467)	0.742		
AJCC stage				
I+II III+IV	Reference 13.043 (8.75-19.442)	<0.001	11.4 (7.463-17.414)	<0.001
TFE3 expression				
Negative Positive	Reference 3.272 (1.936-5.53)	<0.001	2.32 (1.345-4.000)	0.002

**Figure 4:**
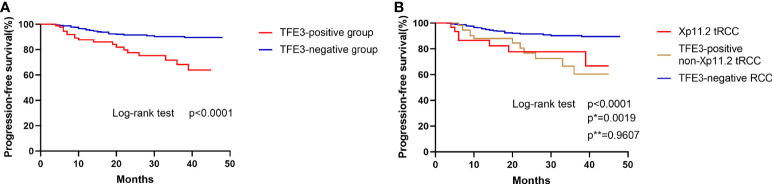
Kaplan–Meier curves for progression-free survival. **(A)** Comparison of progression-free survival between TFE3-positive RCC and TFE3-negative RCC. **(B)** Comparison of progression-free survival among Xp11.2 translocation renal cell carcinoma, TFE3-positive non- Xp11.2 translocation renal cell carcinoma, and TFE3-negative renal cell carcinoma. (p, TFE3-negative vs TFE3-positive non-Xp11.2 translocation RCC; p*, Xp11.2 translocation vs TFE3-negative RCC; p**, Xp11.2 translocation vs TFE3-positive non-Xp11.2 translocation RCC).

To conduct the survival analysis, the patients selected for the current study were divided into two groups (TFE3-positive RCC group and TFE3-negative RCC group). The obtained results of survival analysis revealed that positive expression of TFE3 was correlated with a shorter progression-free survival in patients with RCC (*p*<0.0001, **Figure 4A**). In addition, the patients involved in the present study were divided into three groups (1): TFE3-negative RCC group (2), Xp11.2 translocation RCC group, and (3) TFE3-positive non-Xp11.2 translocation RCC group. After survival analysis study, it was found that the patients in TFE3-negative RCC group were associated with significantly longer progression-free survival time as compared with patients in the other groups in the survival analysis (*p*<0.05). Meanwhile, results of the survival analysis showed that there was no significant difference in progression-free survival between patients in Xp11.2 translocation RCC group and those in the TFE3-positive non-Xp11.2 translocation RCC group (*p*=0.9607, **Figure 4B**).

## Discussion

The current study found that the patients with TFE3-positive RCCs accounted for 11.4% (91 out of 796) of RCCs. However, it was evident that only one-third (31 out of 91) of the TFE3-positive RCCs belonged to the Xp11.2 translocation RCC in the present study. The current study further focused on the clinicopathological characteristics and prognosis of the patients with TFE3-positive RCCs. It was study found that TFE3-positive RCC groups (Xp11.2 translocation RCC and TFE3-positive non-Xp11.2 translocation RCC) showed significantly aggressive clinicopathological characteristics and poor prognosis as compared with the TFE3-negtive RCC group.

Previous studies have regarded TFE3 IHC staining as an important method for diagnosing Xp11.2 translocation RCCs. An early study conducted by Argani et al. (24) showed that the TFE3 IHC staining was highly sensitive and specific for diagnosing Xp11.2 translocation RCCs (97.5 and 99.6%, respectively). With the continuous progress of research on Xp11.2 translocation RCCs, different research studies have also found that not all patients with TFE3-positive RCCs that belongs to the group of Xp11.2 translocation RCCs (12, 23, 25–27). According to the study conducted by Lee et al. (12), 10.2% (31 out of 303) of RCCs expressed positive nuclear TFE3 immunoreactivity and 19.4% (6 out of 31) of TFE3-positive RCCs did not belong to the Xp11.2 translocation RCC group. In the present study, the proportion was increased to 66% (60 out of 91). The difference in the results obtained in the current study as compared with the finding of the previous studies could be attributed to the different definition of TFE3 positive expression. Findings of some previous studies have shown that only moderately or strongly positive expression of TFE3 was considered significant, whereas the weak expression of TFE3 was ignored (7, 12, 15). Notably, RCCs with weak TFE3 staining may also be diagnosed as Xp11.2 translocation RCC using FISH assay and especially in the RCCs with classic histological morphology of Xp11.2 translocation RCC (11, 16).

Based on the previously reported descriptions, the classic morphological presentation feature was defined as tumor cells with abundant eosinophilic or clear cytoplasm with papillary or micropapillary structure, with or without psammoma bodies (16). However, it was found that the classic morphology only presented in half of the TFE3-positive RCCs. Therefore, it was not considered as a significant predictor of rearrangement in TFE3 gene in the previous study (16). The weak expression of nuclear TFE3 was detected in six out of 31 RCCs (19%) Xp11.2 translocation RCCs in the current study and not all RCCs were presented with typical morphology. Currently, TFE3 break-apart FISH assay is currently regarded as the golden standard for the diagnosis of Xp11.2 translocation RCC in clinical practice (8, 16, 28). However, for translocation RCCs with inverted X-chromosome, the RCCs may be presented with equivocal split signals (18–20). Therefore, the results of TFE3 IHC staining are particularly important in such a situation. Previously, we and other found that Xp11.2 translocation RCCs with equivocal FISH results showed varying degrees of positive staining for TFE3 in both the present and the previous studies (19, 20). According to our previous study, a novel NONO-TFE3 dual-fusion FISH assay was developed and the accuracy of this probe validated for diagnosis of NONO-TFE3 RCC (19). In the present study, the NONO-TFE3 fusion was also identified by RNA-sequencing in TFE3-positive RCCs with equivocal split signals. However, the high cost and laborious procedure of RNA-sequencing restricts the wide use of the procedure in the ordinary clinical practice. Therefore, combination of TFE3 IHC with FISH assay is still currently the first choice for diagnosis of the Xp11.2 translocation RCC.

The proportion of TFE3-positive RCCs was 11.4% (91 out of 796) of RCCs in the present study and this was higher than that in the previous studies (9-10.2%) (12, 27). Among the TFE3-positive RCCs in the current study, it was found that approximately one-third (31 out of 91) of the RCCs belong to the Xp11.2 translocation RCC. The incidence of Xp11.2 translocation RCC in the present study was 3.9% (31 out of 796 RCCs), whereas that in previous studies ranged from 1 to 5% among all the RCCs (9–11). It was evident that patients with Xp11.2 translocation RCC in the present study were significantly younger and predominantly female as compared with patients with other RCCs where the patient were significantly older and predominantly male. Meanwhile, the observations were in consonance with those reported in previous studies (8, 10, 29). Xp11.2 translocation RCC often develop lymph node and distant metastasis because of its aggressive characteristics (7, 8, 11, 16). In a separate study, Classe et al. found that the probability of developing lymph node and distant metastasis in Xp11.2 translocation RCCs were 25% (5 out of 20) and 15% (3 out of 20), respectively (11). However, the present study found that six (19.4%) and three (9.7%) patients with Xp11.2 translocation RCC had lymph node metastasis and distant metastasis, respectively. Furthermore, similar probabilities were found in TFE3-positive RCCs, although such probabilities are not as high as that for Xp11.2 translocation RCCs. In the present study, it was found that the probability of lymph node and distant metastasis in the TFE3-positive RCC were 10.9% (10 out of 91 patients) and 6.6% (6 out of 91), respectively, whereas the patient in the TFE3-negative RCC were 1.3% (9 out of 705 patients) and 0.9% (6 out of 105 patients), respectively.

Although some previous studies have suggested that Xp11.2 translocation RCCs tended are of a higher nuclear grade (12, 27, 30), our previous study showed that both the WHO/ISUP and Fuhrman grading system are not suitable for Xp11.2 translocation RCCs (21). Results of the present study showed that the proportion of high nuclear grade was 55% (33 out of 60 patients) and 22% (153 out of 705 patients) in TFE3-positive non-Xp11.2 translocation RCC group and TFE3-negative RCC group, respectively. In addition, the present study evidently found that the proportion of TFE3-positive RCCs with high WHO/ISUP grade or AJCC stage was significantly higher than that of TFE3-negative RCC group. In conclusion, RCCs with positive expression of TFE3 were associated with higher rates of metastasis and higher WHO/ISUP grade as well as AJCC stage.

The findings of the multivariate analysis showed that positive expression of TFE3 was an independent prognostic factor affecting the progression-free survival. According to the expression of TFE3 proteins, the RCCs were divided into two subgroups for survival analysis. Results of the analysis found that the progression-free survival of TFE3-positive RCC group was significantly shorter as compared with that of the TFE3-negative RCC group. When RCCs was classified as TFE3-negative RCC, Xp11.2 translocation RCC, and TFE3-positive non-Xp11.2 translocation RCC groups, it was evident that the TFE3-negative RCC group had a significantly longer progression-free survival as compared with the other groups. Meanwhile, it was noted that there was no significant difference in progression-free survival between Xp11.2 translocation RCC and TFE3-positive non-Xp11.2 translocation RCC groups. Therefore, the present study proposes that expression of TFE3 may be an independent prognostic factor irrespective of the TFE3 translocation states. Furthermore, as long as the RCC has positive expression of TFE3, it may show a worse prognosis compared with the RCC having negative expression of TFE3.

Currently, the reason for TFE3 expression in RCCs could be explained: First, translocation leads to the fusion of the TFE3 gene with several partner genes which results in the overexpression of the fusion proteins (24). Overexpression of the fused of the TFE3 gene facilitates tumor progression and this is also a typical feature of the Xp11.2 translocation RCCs. Second, the presence of the gene amplification may also relate to the expression of TFE3 (27, 31). Previous studies have shown that amplified TFEB tumors express aggressive characteristics, although the gene rearrangement was not observed (16, 32, 33). Third, although it is unknown whether the nuclear localization promotes the expression of TFE3, inactivation of the tumor suppressor gene, FLCN, contributes to the increased TFE3 transcriptional activity and nuclear localization (34). Apart from the described reasons, there are further unknown molecular mechanisms leading to the overexpression of TFE3 in RCC waiting for us to explore. Meanwhile, TFE3, as a member of MiTF family, is involved in the occurrence and development of RCC. Multiple autophagy-associated signaling pathways are regulated by the TFE3 gene, thereby influencing tumor growth (35). The TFE3 protein could inhibit the p21-mediated pRB pathway and activate the P13K/AKT/mTOR pathway, thereby leading to excessive proliferation tumor cells and ultimately causing progression of the tumors (36–38). There is still need for further studies to verify the prognostic value of TFE3 in patients with RCC.

The present study had some limitations. First, the relatively small population of patient and the short patient of follow-up period may affect the accuracy of the obtained results. Second, further molecular detection was not performed on all TFE3-positive RCCs beyond FISH assay, and this could affect the accuracy of the diagnosis conducted in the current study. RNA-sequencing in the present study was only conducted on the RCCs with moderate or strong nuclear TFE3 immunoreactivity because performing RNA-sequencing on every TFE3-positive RCCs would be expensive. Third, the definition of the TFE3-positive RCC remains controversial. Whereby, weak expression of TFE3 is considered insignificant in some studies. However, it was found that the RCC with weak positive expression can be diagnosed as Xp11.2 translocation RCC. Furthermore, even when there is no gene rearrangement, such RCCs have a poor prognosis as compared with the TFE3-negative RCCs.

The FISH assay should be performed in every TFE3-positive RCC. This is because the nuclear expression of TFE3 is not exclusive to the Xp11.2 translocation RCC, but also appears in other types of RCCs. In addition, RNA-sequencing is necessary as a diagnostic test for detection of TFE3 rearrangement. This is for the cases with equivocal split signals, especially with moderate or strong nuclear TFE3 immunoreactivity. Results of the present study demonstrated that the expression of TFE3 in RCCs was significantly associated with higher nuclear grade, tumor stage, and metastasis. Furthermore, it was found that the expression of TFE3 protein in the RCC correlates with the tumor progression and poor prognosis irrespective of the presence of TFE3 translocation.

## Data availability statement

The original contributions presented in the study are included in the article/supplementary material. Further inquiries can be directed to the corresponding author.

## Ethics statement

The studies involving human participants were reviewed and approved by Institutional review board of Nanjing Drum Tower Hospital. Written informed consent for participation was not required for this study in accordance with the national legislation and the institutional requirements.

## Author contributions

XD and WG conceived and designed the study. YC, JP, and WM contributed to the acquisition of data. PZ, MC, and HG supervised the study and review the manuscript. All authors read and approved the final manuscript. All authors contributed to the article and approved the submitted version.

## Conflict of interest

The authors declare that the research was conducted in the absence of any commercial or financial relationships that could be construed as a potential conflict of interest.

## Publisher’s note

All claims expressed in this article are solely those of the authors and do not necessarily represent those of their affiliated organizations, or those of the publisher, the editors and the reviewers. Any product that may be evaluated in this article, or claim that may be made by its manufacturer, is not guaranteed or endorsed by the publisher.
